# Emerging Roles for Lymphatics in Chronic Liver Disease

**DOI:** 10.3389/fphys.2019.01579

**Published:** 2020-01-14

**Authors:** Matthew A. Burchill, Alyssa R. Goldberg, Beth A. Jirón Tamburini

**Affiliations:** ^1^Division of Gastroenterology and Hepatology, Department of Medicine, University of Colorado Anschutz Medical Campus, School of Medicine, Aurora, CO, United States; ^2^Section of Pediatric Gastroenterology, Hepatology and Nutrition, Digestive Health Institute, Children’s Hospital Colorado, Aurora, CO, United States; ^3^Department of Immunology and Microbiology, University of Colorado Anschutz Medical Campus, School of Medicine, Aurora, CO, United States

**Keywords:** lymphatic endothelial cell, liver lymphatics, ascites, chronic liver disease, fatty acids, cholesterol, obesity, portal hypertension

## Abstract

Chronic liver disease (CLD) is a global health epidemic causing ∼2 million deaths annually worldwide. As the incidence of CLD is expected to rise over the next decade, understanding the cellular and molecular mediators of CLD is critical for developing novel therapeutics. Common characteristics of CLD include steatosis, inflammation, and cholesterol accumulation in the liver. While the lymphatic system in the liver has largely been overlooked, the liver lymphatics, as in other organs, are thought to play a critical role in maintaining normal hepatic function by assisting in the removal of protein, cholesterol, and immune infiltrate. Lymphatic growth, permeability, and/or hyperplasia in non-liver organs has been demonstrated to be caused by obesity or hypercholesterolemia in humans and animal models. While it is still unclear if changes in permeability occur in liver lymphatics, the lymphatics do expand in number and size in all disease etiologies tested. This is consistent with the lymphatic endothelial cells (LEC) upregulating proliferation specific genes, however, other transcriptional changes occur in liver LECs that are dependent on the inflammatory mediators that are specific to the disease etiology. Whether these changes induce lymphatic dysfunction or if they impact liver function has yet to be directly addressed. Here, we will review what is known about liver lymphatics in health and disease, what can be learned from recent work on the influence of obesity and hypercholesterolemia on the lymphatics in other organs, changes that occur in LECs in the liver during disease and outstanding questions in the field.

## Introduction

The lymphatic system is comprised of highly permeable capillaries found within the tissue and are required to transport lymph which contains cellular proteins, lipoproteins and lymphocytes (Dixon et al., 2009; Lim et al., 2009; Platt et al., 2013; Hansen et al., 2015; Thomas et al., 2016; Schineis et al., 2019). The capillaries drain into collecting lymphatic vessels (LV) surrounded by lymphatic muscle cells (LMC) that pump the lymph through highly specialized lymphatic valves (Chakraborty et al., 2015). How or if liver disease changes the normal functions of the lymphatics in the liver is not well understood. Here, we will outline current knowledge regarding the liver lymphatic system and lymphatic dysfunction in other organs to highlight underlying questions of how lymphatics may be affected by chronic liver disease (CLD).

## Liver Lymphatics in Health

**FIGURE 1:**
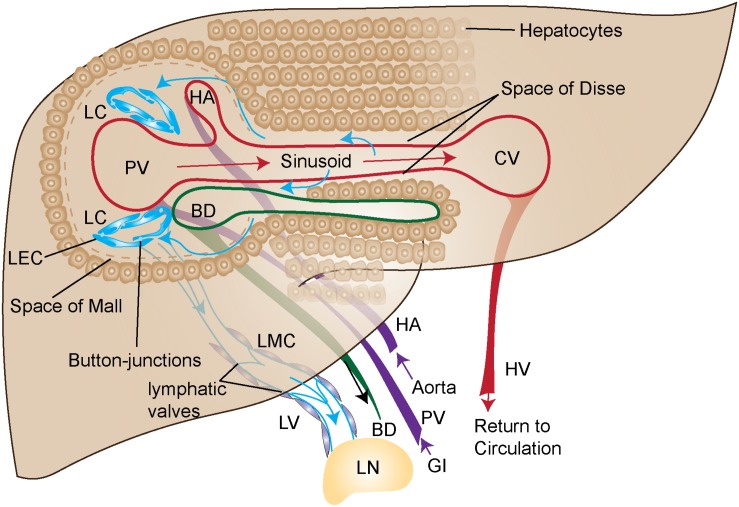
Graphical illustration of liver blood and lymph circulation. Blood (purple arrows) flows into the portal vein (PV) from the gastrointestinal tract (GI) or into hepatic artery (HA) from the aorta. PV and HA as depicted in this figure are branches of the PV and HA. The blood is then transported through the liver sinusoids (red arrows) made up of liver sinusoidal endothelial cells (LSEC) and exits the liver through the central vein (CV) where it returns to circulation via the hepatic vein (HV). The primary source of lymph (blue arrows) travels from the sinusoids, through the Space of Disse, where it enters the Space of Mall and then enters the lymphatic capillaries (LC). The LC are made up of highly permeable button-like junctions that allow cells and protein to easily pass through. Upon entry into the lymphatic capillary the fluid and cells are then propelled through the lymphatic vessels (LV) where lymphatic muscle cells (LMC) pump the lymph toward the draining lymph node (LN). The lymphatic vessels contain valves for unidirectional flow. More than 80% of lymph is transported to portal lymphatic vessels. Superficial and sublobular lymph flow are not shown here.

The liver is the largest contributor to lymph production in the body and accounts for up to 50% of lymph entering the thoracic duct (Tanaka and Iwakiri, 2016). This lymph originates predominantly from the blood vascular system, which is characterized by a portal vein (PV) and hepatic artery (HA) in the portal triad spanning the hepatic lobule to the central vein (CV). Lymphatic structures are found within the portal triad near the PV and bile duct (Figure 1). These are likely lymphatic capillaries (LC) with highly permeable button like junctions and are not surrounded by smooth muscle actin (Tamburini et al., 2019). Fluid from the blood vasculature leaks into the interstitium through highly permeable liver sinusoidal endothelial cells (LSEC). These highly permeable sinusoids are thought to lead to the higher protein content of hepatic lymph as compared to lymph in other organs (Courtice et al., 1962; Witte C.L. et al., 1969; Aukland et al., 1984). Cellular byproducts from hepatocytes, hepatic stellate cells (HSCs) and Kupffer cells also flow into the interstitium.

From the interstitium of the liver, fluid flows into the space of Disse located between the hepatocytes and the blood sinusoids of the liver. The lymphatic fluid then predominantly flows through the space of Mall at the interface of the portal tract and the adjacent hepatocytes. Eighty percent or more of the lymph then flows into LC found along the portal tract (Ritchie et al., 1959) with lymph flow in the same direction as bile and prevented from re-entering the liver by lymphatic valves in downstream collecting vessels (Trutmann and Sasse, 1994). The LC then drain into LV which are surrounded by lymphatic muscle cells (LMC). These LMCs are needed to pump the lymph from the liver into the portal and the celiac lymph nodes (LNs) in mice (Barbier et al., 2012; Zheng et al., 2013; Chakraborty et al., 2015; Figure 1). The portal and celiac LNs in mice correlate to analogous hepatic draining LN clusters in humans (Harrell et al., 2008), from which lymph drains into the cisterna chyli.

In addition to portal lymph there is also sublobular and superficial lymph. Sublobular LV are found along the inferior vena cava (Ohtani and Ohtani, 2008). Superficial lymph drains through various LC toward the periphery of the liver before entering LV draining to regional LNs (Pupulim et al., 2015). These studies describing the normal flow and function of the lymphatics in the liver lay the groundwork for understanding how lymphatic function in the liver could be compromised during disease.

## Liver Lymphatics in Disease

During inflammation, lymphangiogenesis occurs in response to vascular endothelial growth factors (VEGF) expressed by infiltrating macrophages (Kataru et al., 2009). While the precise cell types that produce VEGFC/D to induce lymphangiogenesis in the liver are unknown, several liver resident cells including Kupffer Cells and LSECs have been shown to produce VEGFC/D (Dingle et al., 2018; McGettigan et al., 2019). During end-stage liver disease such as Hepatitis C Viral infection (HCV), Hepatitis B viral infection (HBV), Non-Alcoholic Steatohepatitis (NASH), and Alcoholic Liver Disease (ALD), hepatic lymph production and lymphatic vessel frequency increases in both human patients and animal models (Oikawa et al., 1998; Yamauchi et al., 1998; Yokomori et al., 2010; Tamburini et al., 2019). This same phenomenon of increased lymphatic vessel density, especially in the periportal area, has also been observed in idiopathic portal hypertension and in primary biliary cirrhosis (Oikawa et al., 1998; Yamauchi et al., 2002). The increase in lymphatic vessel density correlates with documented increases in VEGFC/D in the cirrhotic liver (Tugues et al., 2005). In patients with cirrhosis there is also an increase in the volume of lymph fluid draining the liver (Dumont and Mulholland, 1960, 1962; Witte et al., 1980; Granger et al., 1984). However, several studies have reported that the lymph fluid released from the liver, normally rich in protein, has a low protein content in humans (Witte M.H. et al., 1969; Witte et al., 1981) and rats (Barrowman and Granger, 1984) with cirrhosis. These findings suggest that despite the increase in frequency of lymphatics during disease, there may be a decrease in lymphatic permeability which prevents the proper removal of inflammatory mediators and cells from the liver. Despite these findings, it has yet to be determined if the lymphatics in the liver are functioning properly or if these changes in lymph content occur as a consequence of other disease-dependent mechanisms.

## Liver Lymphatic Endothelial Cells

The network of liver LV develops in the prenatal period (Paupert et al., 2011) by differentiation from blood vasculature through the expression of *Prox1* (Wigle et al., 2002). *Prox1*, the transcription factor required for lineage commitment of lymphatic endothelial cells (LEC) (Wigle and Oliver, 1999; Petrova et al., 2002), is also expressed by hepatocytes (Dudas et al., 2004, 2006; Tamburini et al., 2019). As such, the lymphatic vasculature in the liver lacks a single specific marker, however, LV can specifically be imaged using PDPN (D2-40) in humans. The D2-40 stain in humans is fairly specific for lymphatic vasculature and not the vascular endothelium nor liver epithelium (Yokomori et al., 2010; Tamburini et al., 2019). However, this is not as straight forward in mice as cholangiocytes are positive for the 8.1.1 anti-mouse PDPN clone (Finlon et al., 2019). Other common lymphatic endothelial markers include LYVE-1, but in the liver LYVE-1 is also expressed on LSECs and macrophages (Mouta Carreira et al., 2001). VEGFR3, which is an essential tyrosine kinase receptor in vascular endothelial growth, is also expressed by other cells in the liver including LSECs and cholangiocytes (Gaudio et al., 2006; Tamburini et al., 2019). Other less common lymphatic markers include CCL21, MMR1, desmoplakin, and integrin α9 (Petrova et al., 2002), all of which are also expressed by other liver resident cells and thus distinguishing liver lymphatics, especially in mouse models, has been difficult. Table 1 outlines common markers and overlap among other liver specific cell populations in mice. Combinations of these markers can be used for the identification of LEC in the liver by flow cytometry and immunofluorescence as described in Finlon et al. (2019).

**TABLE 1:** Marker distribution that can be used to distinguish lymphatic endothelial cells (LEC) (Iwakiri, 2016; Finlon et al., 2019; Tamburini et al., 2019) from hepatocytes (Dudas et al., 2006; Goto et al., 2017), cholangiocytes (Gaudio et al., 2006; Li et al., 2013; Lua et al., 2014, 2016), liver sinusoidal endothelial cells (LSEC) (Schrage et al., 2008; Ding et al., 2010; Strauss et al., 2017), and portal endothelial cells (PEC) (Schrage et al., 2008) for flow cytometry or immunofluorescence.

	**Hepatocytes**	**Cholangiocytes**	**LSEC**	**PEC**	**LEC**
PDPN	−	+	−	−	+
Prox1	+	−	−	−	+
LYVE-1	−	−	+	−	+
Vegfr3/Flt4	−	+	+	+	+
CK19	−	+	−	−	−
Albumin	+	−	−	−	−
CCL21	−	−	±	−	+
CD146	−	−	+	+	−/low

To address this, our recent studies evaluated liver LECs by single cell mRNA sequencing. However, we were unable to uncover a marker that is specific to LECs in the liver (Tamburini et al., 2019). We were able to differentiate LECs from other cells in the liver through the combined expression of *PDPN*, *LYVE1*, *PROX1*, VEGFR3 (*FLT4*), and *CCL21*. While the expression of these markers individually are not unique to the LECs in the liver, LECs are the only population of cells that express all of these markers together. Using these cell markers, we identified that the LECs within a cirrhotic liver are actively proliferating, consistent with increased lymphatic vessel density (Tamburini et al., 2019). We also discovered that LECs in disease upregulate pathways involved in the production of superoxide and reactive oxygen species consistent with the LECs responding to liver inflammation (Tamburini et al., 2019).

The transcriptional profile of LECs in the liver is dependent on disease etiology. Specifically, during chronic HCV infection the LECs upregulate genes associated with responses to increased interferon in the liver through Signal Transducer and Activator of Transcription 1 (STAT1), among others. In contrast, LECs isolated from the livers of patients with obesity associated NASH, upregulated genetic programs related to downstream cholesterol signaling and IL13 signaling, potentially through the upregulation of CD36 and p38 mitogen activated protein kinase (MAPK) (Tamburini et al., 2019). This study was the first to report the transcriptional profile of LECs isolated from healthy or cirrhotic human livers. Whether these transcriptional differences cause lymphatic dysfunction can only be inferred based on responses in other tissues.

## Obesity and Lymphatics

In recent years obesity has been implicated as the cause of many diseases including cardiovascular disease, diabetes, and NASH. As excess stress on the body caused by obesity results in a type of chronic inflammatory state in the liver, heart and other organs it is perhaps not surprising that the lymphatic system is also affected. In an elegant report, Garcia Nores et al. (2016) demonstrated that obesity is a root cause of lymphatic dysfunction in the skin. Additionally, recent reports suggest that peri-lymphatic inflammation results in lymphatic structures that are functionally impaired causing defective interstitial fluid drainage and decreased dendritic cell (DC) migration (Weitman et al., 2013). Lymphatic dysfunction together with inflammation caused by disease can worsen inflammation and disease pathology (Cuzzone et al., 2014). In these studies, it was demonstrated that stearic acid, a long chain fatty acid commonly associated with obese tissues was highly toxic to LECs *in vitro*. This toxicity could be prevented by increasing phosphatidylinositol-triphosphate (PIP3) kinase signaling (Platt et al., 2013; Hansen et al., 2015; Thomas et al., 2016), presumably through phosphatase and tensin homolog (PTEN) activity (Garcia Nores et al., 2016). PTEN can be activated by VEGFR3 tyrosine kinase receptor activity (Garcia Nores et al., 2016). Furthermore, the peri-lymphatic inflammation seen was described to be a result of CD4^+^ T cell and/or macrophage activity as lymphatic defects were ameliorated in CD4^+^ T cell depleted mice fed a high fat diet (Torrisi et al., 2016); and macrophage crown like structures were observed in close proximity to defective LV (Garcia Nores et al., 2016). Intriguingly, lymphatic dysfunction, inflammation and iNOS activity can all be reversed by weight loss (Nitti et al., 2016). This is perhaps not too surprising as many of the co-morbidities associated with obesity are also resolved after weight loss (Magkos et al., 2016). Finally, in *Prox1* heterozygous mice, a genetic model of lymphatic dysfunction (Wigle and Oliver, 1999; Harvey et al., 2005; Rutkowski et al., 2006), the LV become ruptured and “leaky” (Escobedo and Oliver, 2017). The ruptured and leaky lymphatics to lead to the accumulation of free fatty acids in the surrounding adipose tissue and increased adipogenesis (Harvey et al., 2005; Escobedo et al., 2016). These findings suggest that obesity not only drives lymphatic dysfunction, but that lymphatic dysfunction can drive obesity. Thus, in the setting of obesity associated liver disease it is possible that increased steatosis and the resulting inflammation, steatohepatitis, may occur around LC or vessels. This peri-lymphatic inflammation could cause toxicity or changes in signaling pathways in LECs that lead to decreased lymphatic integrity and increased adipogenesis in the surrounding tissues.

## VEGF and Chylomicron Transport

The lymphatics within the intestinal villi, lacteals, are required for dietary fat uptake. The lacteals have highly permeable “button-like” junctions that allow for the acquisition of dietary fat, in the form of chylomicrons. As such, genetic deletion of *Neuropillin 1* together with *Vascular endothelial growth factor receptor 1* in endothelial cells, resulted in a decrease of button-like junctions and increase in zipper-like junctions in intestinal lacteals that led to the malabsorption of chylomicrons (Zhang et al., 2018). Additionally, complete loss of VEGFC expression by LECs in adult mice led to severe defects in lacteal regeneration and compromised dietary fat absorption (Nurmi et al., 2015). VEGFD (a VEGFR3 ligand) has also been shown to participate in the removal of chylomicron remnants from the blood (Tirronen et al., 2018). In a recent report, Tirronen et al. (2018) found that loss of VEGFD resulted in the downregulation of syndecan 1 (SDC1) on the sinusoidal surface of hepatocytes (Tirronen et al., 2018). SDC1 is a receptor for APO-B found in chylomicron remnants. APO-B is also important for hepatic uptake of tri-glyceride rich lipoproteins. These new findings demonstrate that VEGFD can mediate crosstalk between the LSECs and hepatocytes for chylomicron remnant removal from the blood. However, what contribution loss of VEGFD had to liver lymphangiogenesis or reverse cholesterol transport was not investigated. This work suggests an intimate crosstalk between the vascular and lymphatic endothelium with liver and intestine specific cells. This crosstalk appears to have both specific and non-specific consequences for lipid and cholesterol metabolism, lymphatic growth and differentiation, and/or transport of fatty acids and chylomicrons throughout the body.

## Hypercholesterolemia and Lymphatics

In addition to the interplay between obesity and lymphatics, recent studies have highlighted the role of lymphatics in reverse cholesterol transport and the impact of hypercholesterolemia on lymphatic function. Reverse cholesterol transport is the process of the removal of cholesterol from peripheral tissues back to the liver where it is excreted through the bile (Brufau et al., 2011). This process requires cholesterol to be received by the lymphatic vasculature before entering into the blood stream at the thoracic duct (Huang et al., 2015). Recent reports have suggested that LV are not just passive transporters of cholesterol, but instead may be actively involved in the regulation of fatty acids and cholesterol transport from the tissue similar to how lymphatics regulate immune cell transport (Baluk et al., 2007; Pflicke and Sixt, 2009; Lim et al., 2013; Martel et al., 2013). In light of this, several studies have recently been done to understand the impact of cholesterol on lymphatic function. Many of these studies have been performed in *ApoE*−/− mice fed a high fat diet. These mice have exceptionally high levels of cholesterol and LV are more tortuous, dilated, and have areas of smooth muscle cell coverage that are irregular (Lim et al., 2009). As a result, using lymphocyntigraphy and DC migration assays, these LV were functionally impaired in their ability to transport fluid from the tissue or DCs to the LN and this could be rescued by treatment with a cholesterol reducing agent, ezetimibe (Lim et al., 2013). Hypercholesterolemia also led to increased levels of cholesterol in the tissues and LNs which could be rescued by VEGFC or VEGFD treatment (Lim et al., 2013). Further, loss of the cholesterol receptor SR-BI completely ablated cholesterol transport through the lymph (Lim et al., 2013). Finally, it was shown that hypercholesterolemia increased LN cellularity by disrupting normal lymphatic structures (Tay et al., 2019). This was due to decreased S1P secretion into the lymph, decreased *Sphk1* transcript (which catalyzes the biosynthesis of SIP) by LECs and increased *Spgl1* transcript which degrades S1P thereby decreasing lymphocyte egress from the LN (Tay et al., 2019). The effect of cholesterol on LV has yet to be fully understood, but could have a major impact on lymphatic function across systems resulting in defects in lymphocyte trafficking, cholesterol or triglyceride accumulation in the tissue, chylomicron uptake or chylomicron remnant removal. Cholesterol and oxidized cholesterol species are increased in the livers of patients with CLD, thus understanding the impact of cholesterol on lymphatics is essential for understanding lymphatic function in the liver during disease.

To gain insight into specialized roles for liver LECs during disease our recent report examined how LECs respond to cholesterol species retained in the liver during disease, such as highly oxidized low density lipoprotein (OX-LDL). While OX-LDL is a key player in the pathogenesis of atherosclerosis (Di Pietro et al., 2016; Gao and Liu, 2017), it has only recently been appreciated that OX-LDL can contribute to the progression of liver disease. OX-LDL accumulates as a result of free radicals generated by inflammation and can directly induce liver injury (Zhang et al., 2014). Further, levels of OX-LDL are elevated in patients with NASH (Ampuero et al., 2016), HCV (Nakhjavani et al., 2011), ALD (Alho et al., 2004; Schroder et al., 2006), and cholestasis (Comert et al., 2004; Karadeniz et al., 2008). This increased OX-LDL in the pathogenesis of CLD is consistent with our transcriptional data from LECs from livers of people with NASH, in that pathways involved in free radical scavenging, IL13 signaling and genes involved in cholesterol sensing were upregulated (Tamburini et al., 2019). In our studies, OX-LDL induced the production IL13 by LECs in livers of mice but not LN LECs and in contrast to the interferon inducing toll-like receptor (TLR) agonist, polyI:C (Tamburini et al., 2019). LECs have been shown to produce, IL-1β, IL-6, IL-7, IL-8, and iNOS during inflammation and TGFβ at baseline (Card et al., 2014), however, IL-13 production by LECs had yet to be reported. These findings were interesting as recent evidence demonstrated that IL4 and IL13 can reduce *Prox1* expression (Savetsky et al., 2015; Shin et al., 2015). In addition to the expression of IL13, LECs are one of the few cell types to express both IL13Rα1 and IL13Rα2, suggesting that LECs have the ability to both produce and respond to IL13 (Heng et al., 2008). When OX-LDL was administered to human LECs *in vitro*, *Prox1* and *Vegfr3* expression were decreased (Tamburini et al., 2019), similar to what was seen when LECs were treated with recombinant IL13 (Savetsky et al., 2015; Shin et al., 2015). This loss of *Prox1* could significantly affect lymphatic differentiation as in an elegant paper by Wong et al. (2017), it was demonstrated that LECs upregulate fatty acid oxidation (FAO) in a positive feedback loop that that requires PROX1, p300 and CPT1a to promote lymphatic growth and lymphatic specific chromatin modifications. These findings are important as we begin to investigate how OX-LDL and/or IL13 could manipulate the function of LEC and also how the metabolic state of the LEC can be the consequence and/or cause of chronic disease states. This is especially intriguing in the liver as blocking IL-13 can reduce liver fibrosis-at least in a Schistosoma infection model (Chiaramonte et al., 2001). Thus, understanding how different inflammatory mediators within the liver affect lymphatic function will be important for developing novel and specific therapeutic targets for liver lymphatics.

## Bypass of First Pass Metabolism in the Liver by Targeting Lymphatics

Another recent and intriguing area of research regarding lymphatics and the liver is currently being explored within the pharmacologic community. First pass metabolism is a process in which oral drug administration results in drug metabolism in the liver. When the drug passes directly from the intestine into the hepatic PV it is rapidly cleared in the liver via drug metabolizing enzymes, transporters and secretion through the bile (Andrews et al., 2017; Argikar et al., 2017; Mariappan et al., 2017; Miron et al., 2017). This rapid clearance of drugs decreases therapeutic efficacy of the orally administered drug. To circumvent these issues, the lymphatic system is being targeted to bypass first pass metabolism by the liver. As stated above, the lacteals are required for dietary fat uptake from enterocytes in the form of chylomicrons. Through microemulsion formulations of lipids (Khoo et al., 2003), self-microemulsifying drug delivery systems (SMEDDS) (Li et al., 2017), chemical modification with fatty acyl groups/esterification (Borkar et al., 2015, 2016) and other lipid formulations (Han et al., 2015), several groups have targeted the lacteals for oral drug uptake. Targeting lymphatic uptake in the gut results in both localized and systemic drug delivery by bypassing the liver. This method for drug delivery has many potential uses, of which a particularly intriguing area of research is to improve delivery of drugs that modulate immune function. To this end, delivery of lipophilic alkyl derivatives or triglyceride derivatives of the immunosuppressive drug mycophenolic acid (MPA) caused MPA to enter into the lymphatic system, pass into the mesentery and ultimately into the mesenteric LNs to the targeted immune cells (Han et al., 2014). These studies demonstrate a novel way for specifically reaching the lymphatic and the immune system with lipophilic drugs. This is important as we negotiate new ways to manipulate lymphatic or immune function in different organ systems.

## Conclusion and Future Goals

As we move forward into understanding differences in LECs based on their tissue microenvironment we are likely to come across novel and specific pathways that could be targeted for disease therapeutics. Of particular interest are atherosclerosis, inflammatory bowel disease, or CLD in which LC and vessels are important for the clearance of inflammatory infiltrate, protein, cholesterol and triglycerides from the tissue. VEGF-C and VEGF-D have pleiotropic effects, but could be developed for use as recombinant proteins, agonistic antibodies or small molecules to potentiate lymphatic growth and/or function across the board to improve organ health. Similarly, the mTOR inhibitor, sirolimus, has been shown to limit downstream VEGFR3 signaling thereby aiding in diseases where lymphatic malformations occur (Baluk et al., 2017; Ozeki and Fukao, 2019; Ozeki et al., 2019a,b,c). Presumably, based on preclinical models of myocardial infarction promoting lymphangiogenesis could improve organ function (Klotz et al., 2015). However, based on recent findings it may be more relevant to focus our studies on understanding the specific contributors to lymphatic dysfunction, such as OX-LDL, and target the removal of those inflammatory mediators instead. In parallel, it will be important to understand downstream signaling pathways in LECs caused by inflammatory mediators or inflammatory cells with the end goal of maintaining lymphatic function through the prevention of signals induced by the inflammation. In the end, it is important for us to understand differences in lymphatics in each organ system, the specific stressors these cells encounter and how they can be targeted using lipophilic drug derivatives. At this time it is fairly evident that a common function of lymphatics across systems is to promote lipid and protein homeostasis to maintain organ health.

## Author Contributions

MB, AG, and BT all contributed to the conception and writing of the manuscript.

## Conflict of Interest

The authors declare that the research was conducted in the absence of any commercial or financial relationships that could be construed as a potential conflict of interest.

## References

[B1] Alho H. Sillanaukee P. Kalela A. Jaakkola O. Laine S. Nikkari S. T. (2004). Alcohol misuse increases serum antibodies to oxidized LDL and C-reactive protein. *Alcohol. Alcohol.* 39 312–315. 10.1093/alcalc/agh059 15208162

[B2] Ampuero J. Ranchal I. Gallego-Duran R. Pareja M. J. Del Campo J. A. Pastor-Ramirez H. (2016). Oxidized low-density lipoprotein antibodies/high-density lipoprotein cholesterol ratio is linked to advanced non-alcoholic fatty liver disease lean patients. *J. Gastroenterol. Hepatol.* 31 1611–1618. 10.1111/jgh.13335 26946071

[B3] Andrews L. M. Li Y. De Winter B. C. M. Shi Y. Y. Baan C. C. Van Gelder T. (2017). Pharmacokinetic considerations related to therapeutic drug monitoring of tacrolimus in kidney transplant patients. *Expert Opin. Drug Metab. Toxicol.* 13 1225–1236. 10.1080/17425255.2017.1395413 29084469

[B4] Argikar U. A. Dumouchel J. L. Dunne C. E. Bushee A. J. (2017). Ocular non-P450 oxidative, reductive, hydrolytic, and conjugative drug metabolizing enzymes. *Drug Metab. Rev.* 49 372–394. 10.1080/03602532.2017.1322609 28438049

[B5] Aukland K. Kramer G. C. Renkin E. M. (1984). Protein concentration of lymph and interstitial fluid in the rat tail. *Am. J. Physiol.* 247(1 Pt 2), H74–H79. 6742214 10.1152/ajpheart.1984.247.1.H74

[B6] Baluk P. Fuxe J. Hashizume H. Romano T. Lashnits E. Butz S. (2007). Functionally specialized junctions between endothelial cells of lymphatic vessels. *J. Exp. Med.* 204 2349–2362. 10.1084/jem.20062596 17846148 PMC2118470

[B7] Baluk P. Yao L. C. Flores J. C. Choi D. Hong Y. K. McDonald D. M. (2017). Rapamycin reversal of VEGF-C-driven lymphatic anomalies in the respiratory tract. *JCI Insight.* 2:16. 10.1172/jci.insight.90103 28814666 PMC5621869

[B8] Barbier L. Tay S. S. McGuffog C. Triccas J. A. McCaughan G. W. Bowen D. G. (2012). Two lymph nodes draining the mouse liver are the preferential site of DC migration and T cell activation. *J. Hepatol.* 57 352–358. 10.1016/j.jhep.2012.03.023 22542491

[B9] Barrowman J. A. Granger D. N. (1984). Effects of experimental cirrhosis on splanchnic microvascular fluid and solute exchange in the rat. *Gastroenterology* 87 165–172. 10.1016/0016-5085(84)90140-9 6724260

[B10] Borkar N. Chen Z. Saaby L. Mullertz A. Hakansson A. E. Schonbeck C. (2016). Apomorphine and its esters: differences in Caco-2 cell permeability and chylomicron affinity. *Int. J. Pharm.* 509 499–506. 10.1016/j.ijpharm.2016.06.010 27282537

[B11] Borkar N. Li B. Holm R. Hakansson A. E. Mullertz A. Yang M. (2015). Lipophilic prodrugs of apomorphine I: preparation, characterisation, and in vitro enzymatic hydrolysis in biorelevant media. *Eur. J. Pharm. Biopharm.* 89 216–223. 10.1016/j.ejpb.2014.12.014 25513957

[B12] Brufau G. Groen A. K. Kuipers F. (2011). Reverse cholesterol transport revisited: contribution of biliary versus intestinal cholesterol excretion. *Arterioscler. Thromb. Vasc. Biol.* 31 1726–1733. 10.1161/ATVBAHA.108.181206 21571685

[B13] Card C. M. Yu S. S. Swartz M. A. (2014). Emerging roles of lymphatic endothelium in regulating adaptive immunity. *J. Clin. Invest.* 124 943–952. 10.1172/JCI73316 24590280 PMC3938271

[B14] Chakraborty S. Davis M. J. Muthuchamy M. (2015). Emerging trends in the pathophysiology of lymphatic contractile function. *Semin. Cell Dev. Biol.* 38 55–66. 10.1016/j.semcdb.2015.01.005 25617600 PMC4397138

[B15] Chiaramonte M. G. Cheever A. W. Malley J. D. Donaldson D. D. Wynn T. A. (2001). Studies of murine schistosomiasis reveal interleukin-13 blockade as a treatment for established and progressive liver fibrosis. *Hepatology* 34 273–282. 10.1053/jhep.2001.26376 11481612

[B16] Comert M. Tekin I. O. Acikgoz S. Ustundag Y. Ucan B. H. Acun Z. (2004). Experimental bile-duct ligation resulted in accumulation of oxidized low-density lipoproteins in BALB/c mice liver. *J. Gastroenterol. Hepatol.* 19 1052–1057. 10.1111/j.1440-1746.2004.03400.x 15304124

[B17] Courtice F. C. Woolley G. Garlick D. G. (1962). The transference of macromolecules from plasma to lymph in the liver. *Aust. J. Exp. Biol. Med. Sci.* 40 111–119. 13881756 10.1038/icb.1962.14

[B18] Cuzzone D. A. Weitman E. S. Albano N. J. Ghanta S. Savetsky I. L. Gardenier J. C. (2014). IL-6 regulates adipose deposition and homeostasis in lymphedema. *Am. J. Physiol. Heart Circ. Physiol.* 306 H1426–H1434. 10.1152/ajpheart.01019.2013 24633552 PMC4024716

[B19] Di Pietro N. Formoso G. Pandolfi A. (2016). Physiology and pathophysiology of oxLDL uptake by vascular wall cells in atherosclerosis. *Vascul Pharmacol.* 84 1–7. 10.1016/j.vph.2016.05.013 27256928

[B20] Ding B. S. Nolan D. J. Butler J. M. James D. Babazadeh A. O. Rosenwaks Z. (2010). Inductive angiocrine signals from sinusoidal endothelium are required for liver regeneration. *Nature* 468 310–315. 10.1038/nature09493 21068842 PMC3058628

[B21] Dingle A. M. Yap K. K. Gerrand Y. W. Taylor C. J. Keramidaris E. Lokmic Z. (2018). Characterization of isolated liver sinusoidal endothelial cells for liver bioengineering. *Angiogenesis* 21 581–597. 10.1007/s10456-018-9610-0 29582235

[B22] Dixon J. B. Raghunathan S. Swartz M. A. (2009). A tissue-engineered model of the intestinal lacteal for evaluating lipid transport by lymphatics. *Biotechnol. Bioeng.* 103 1224–1235. 10.1002/bit.22337 19396808 PMC3132564

[B23] Dudas J. Elmaouhoub A. Mansuroglu T. Batusic D. Tron K. Saile B. (2006). Prospero-related homeobox 1 (Prox1) is a stable hepatocyte marker during liver development, injury and regeneration, and is absent from “oval cells”. *Histochem Cell Biol.* 126 549–562. 10.1007/s00418-006-0191-4 16770575

[B24] Dudas J. Papoutsi M. Hecht M. Elmaouhoub A. Saile B. Christ B. (2004). The homeobox transcription factor Prox1 is highly conserved in embryonic hepatoblasts and in adult and transformed hepatocytes, but is absent from bile duct epithelium. *Anat. Embryol.* 208 359–366. 15232737 10.1007/s00429-004-0403-4

[B25] Dumont A. E. Mulholland J. H. (1960). Flow rate and composition of thoracic-duct lymph in patients with cirrhosis. *N. Engl. J. Med.* 263 471–474. 10.1056/nejm196009082631001 13818600

[B26] Dumont A. E. Mulholland J. H. (1962). Alterations in thoracic duct lymph flow in hepatic cirrhosis: significance in portal hypertension. *Ann. Surg.* 156 668–675. 17859709 10.1097/00000658-196210000-00013 PMC1466254

[B27] Escobedo N. Oliver G. (2017). The lymphatic vasculature: its role in adipose metabolism and obesity. *Cell Metab.* 26 598–609. 10.1016/j.cmet.2017.07.020 28844882 PMC5629116

[B28] Escobedo N. Proulx S. T. Karaman S. Dillard M. E. Johnson N. Detmar M. (2016). Restoration of lymphatic function rescues obesity in Prox1-haploinsufficient mice. *JCI Insight.* 1:e85096. 10.1172/jci.insight.85096 26973883 PMC4786184

[B29] Finlon J. M. Burchill M. A. Tamburini B. A. J. (2019). Digestion of the murine liver for a flow cytometric analysis of lymphatic endothelial cells. *J. Vis. Exp.* 143:e58621. 10.3791/58621 30663671 PMC6350926

[B30] Gao S. Liu J. (2017). Association between circulating oxidized low-density lipoprotein and atherosclerotic cardiovascular disease. *Chronic Dis. Transl. Med.* 3 89–94. 10.1016/j.cdtm.2017.02.008 29063061 PMC5627698

[B31] Garcia Nores G. D. Cuzzone D. A. Albano N. J. Hespe G. E. Kataru R. P. Torrisi J. S. (2016). Obesity but not high-fat diet impairs lymphatic function. *Int. J. Obes.* 40 1582–1590. 10.1038/ijo.2016.96 27200507 PMC5050064

[B32] Gaudio E. Barbaro B. Alvaro D. Glaser S. Francis H. Ueno Y. (2006). Vascular endothelial growth factor stimulates rat cholangiocyte proliferation via an autocrine mechanism. *Gastroenterology* 130 1270–1282. 10.1053/j.gastro.2005.12.034 16618418

[B33] Goto T. Elbahrawy A. Furuyama K. Horiguchi M. Hosokawa S. Aoyama Y. (2017). Liver-specific Prox1 inactivation causes hepatic injury and glucose intolerance in mice. *FEBS Lett.* 591 624–635. 10.1002/1873-3468.12570 28129664

[B34] Granger D. N. Perry M. A. Kvietys P. R. Taylor A. E. (1984). Capillary and interstitial forces during fluid absorption in the cat small intestine. *Gastroenterology* 86 267–273. 10.1016/0016-5085(84)90410-4 6690353

[B35] Han S. Hu L. Quach T. Simpson J. S. Trevaskis N. L. Porter C. J. (2015). Profiling the role of deacylation-reacylation in the lymphatic transport of a triglyceride-mimetic prodrug. *Pharm. Res.* 32 1830–1844. 10.1007/s11095-014-1579-9 25446770

[B36] Han S. Quach T. Hu L. Wahab A. Charman W. N. Stella V. J. (2014). Targeted delivery of a model immunomodulator to the lymphatic system: comparison of alkyl ester versus triglyceride mimetic lipid prodrug strategies. *J. Control Release* 177 1–10. 10.1016/j.jconrel.2013.12.031 24398334

[B37] Hansen K. C. D’Alessandro A. Clement C. C. Santambrogio L. (2015). Lymph formation, composition and circulation: a proteomics perspective. *Int. Immunol.* 27 219–227. 10.1093/intimm/dxv012 25788586

[B38] Harrell M. I. Iritani B. M. Ruddell A. (2008). Lymph node mapping in the mouse. *J. Immunol. Methods.* 332 170–174. 10.1016/j.jim.2007.11.012 18164026 PMC2342937

[B39] Harvey N. L. Srinivasan R. S. Dillard M. E. Johnson N. C. Witte M. H. Boyd K. (2005). Lymphatic vascular defects promoted by Prox1 haploinsufficiency cause adult-onset obesity. *Nat. Genet.* 37 1072–1081. 10.1038/ng1642 16170315

[B40] Heng T. S. Painter M. W. Immunological Genome and Project Consortium (2008). The Immunological Genome Project: networks of gene expression in immune cells. *Nat. Immunol.* 9 1091–1094. 10.1038/ni1008-1091 18800157

[B41] Huang L. H. Elvington A. Randolph G. J. (2015). The role of the lymphatic system in cholesterol transport. *Front. Pharmacol.* 6:182. 10.3389/fphar.2015.00182 26388772 PMC4557107

[B42] Iwakiri Y. (2016). The lymphatic system: a new frontier in hepatology. *Hepatology* 64 706–707. 10.1002/hep.28650 27228259 PMC4992465

[B43] Karadeniz G. Acikgoz S. Tekin I. O. Tascylar O. Gun B. D. Comert M. (2008). Oxidized low-density-lipoprotein accumulation is associated with liver fibrosis in experimental cholestasis. *Clinics* 63 531–540. 18719767 10.1590/S1807-59322008000400020 PMC2664132

[B44] Kataru R. P. Jung K. Jang C. Yang H. Schwendener R. A. Baik J. E. (2009). Critical role of CD11b+ macrophages and VEGF in inflammatory lymphangiogenesis, antigen clearance, and inflammation resolution. *Blood* 113 5650–5659. 10.1182/blood-2008-09-176776 19346498

[B45] Khoo S. M. Shackleford D. M. Porter C. J. Edwards G. A. Charman W. N. (2003). Intestinal lymphatic transport of halofantrine occurs after oral administration of a unit-dose lipid-based formulation to fasted dogs. *Pharm Res.* 20 1460–1465. 14567642 10.1023/a:1025718513246

[B46] Klotz L. Norman S. Vieira J. M. Masters M. Rohling M. Dubé K. N. (2015). Cardiac lymphatics are heterogeneous in origin and respond to injury. *Nature* 522:62. 10.1038/nature14483 25992544 PMC4458138

[B47] Li F. Hu R. Wang B. Gui Y. Cheng G. Gao S. (2017). Self-microemulsifying drug delivery system for improving the bioavailability of huperzine A by lymphatic uptake. *Acta Pharm. Sin. B* 7 353–360. 10.1016/j.apsb.2017.02.002 28540173 PMC5430757

[B48] Li Y. Wang J. Asahina K. (2013). Mesothelial cells give rise to hepatic stellate cells and myofibroblasts via mesothelial-mesenchymal transition in liver injury. *Proc. Nat. Acad. Sci. U.S.A.* 110 2324–2329. 10.1073/pnas.1214136110 23345421 PMC3568296

[B49] Lim H. Y. Rutkowski J. M. Helft J. Reddy S. T. Swartz M. A. Randolph G. J. (2009). Hypercholesterolemic mice exhibit lymphatic vessel dysfunction and degeneration. *Am. J. Pathol.* 175 1328–1337. 10.2353/ajpath.2009.080963 19679879 PMC2731150

[B50] Lim H. Y. Thiam C. H. Yeo K. P. Bisoendial R. Hii C. S. McGrath K. C. (2013). Lymphatic vessels are essential for the removal of cholesterol from peripheral tissues by SR-BI-mediated transport of HDL. *Cell Metab.* 17 671–684. 10.1016/j.cmet.2013.04.002 23663736

[B51] Lua I. James D. Wang J. Wang K. S. Asahina K. (2014). Mesodermal mesenchymal cells give rise to myofibroblasts, but not epithelial cells, in mouse liver injury. *Hepatology* 60 311–322. 10.1002/hep.27035 24488807 PMC4077971

[B52] Lua I. Li Y. Zagory J. A. Wang K. S. French S. W. Sevigny J. (2016). Characterization of hepatic stellate cells, portal fibroblasts, and mesothelial cells in normal and fibrotic livers. *J. Hepatol.* 64 1137–1146. 10.1016/j.jhep.2016.01.010 26806818 PMC4834254

[B53] Magkos F. Fraterrigo G. Yoshino J. Luecking C. Kirbach K. Kelly S. C. (2016). Effects of moderate and subsequent progressive weight loss on metabolic function and adipose tissue biology in Humans with Obesity. *Cell Metab.* 23 591–601. 10.1016/j.cmet.2016.02.005 26916363 PMC4833627

[B54] Mariappan T. T. Shen H. Marathe P. (2017). Endogenous biomarkers to assess drug-drug interactions by drug transporters and enzymes. *Curr. Drug Metab.* 18 757–768. 10.2174/1389200218666170724110818 28738769

[B55] Martel C. Li W. Fulp B. Platt A. M. Gautier E. L. Westerterp M. (2013). Lymphatic vasculature mediates macrophage reverse cholesterol transport in mice. *J. Clin. Invest.* 123 1571–1579. 10.1172/JCI63685 23524964 PMC3613904

[B56] McGettigan B. McMahan R. Orlicky D. Burchill M. Danhorn T. Francis P. (2019). Dietary lipids differentially shape nonalcoholic steatohepatitis progression and the transcriptome of kupffer cells and infiltrating macrophages. *Hepatology* 70 67–83. 10.1002/hep.30401 30516830 PMC6923128

[B57] Miron A. Aprotosoaie A. C. Trifan A. Xiao J. (2017). Flavonoids as modulators of metabolic enzymes and drug transporters. *Ann. N. Y. Acad. Sci.* 1398 152–167. 10.1111/nyas.13384 28632894

[B58] Mouta Carreira C. Nasser S. M. di Tomaso E. Padera T. P. Boucher Y. Tomarev S. I. (2001). LYVE-1 is not restricted to the lymph vessels: expression in normal liver blood sinusoids and down-regulation in human liver cancer and cirrhosis. *Cancer Res.* 61 8079–8084. 11719431

[B59] Nakhjavani M. Mashayekh A. Khalilzadeh O. Asgarani F. Morteza A. Omidi M. (2011). Oxidized low-density lipoprotein is associated with viral load and disease activity in patients with chronic hepatitis C. *Clin. Res. Hepatol. Gastroenterol.* 35 111–116. 10.1016/j.clinre.2010.11.001 21809486

[B60] Nitti M. D. Hespe G. E. Kataru R. P. Garcia Nores G. D. Savetsky I. L. Torrisi J. S. (2016). Obesity-induced lymphatic dysfunction is reversible with weight loss. *J. Physiol.* 594 7073–7087. 10.1113/JP273061 27619475 PMC5134379

[B61] Nurmi H. Saharinen P. Zarkada G. Zheng W. Robciuc M. R. Alitalo K. (2015). VEGF-C is required for intestinal lymphatic vessel maintenance and lipid absorption. *EMBO Mol. Med.* 7 1418–1425. 10.15252/emmm.201505731 26459520 PMC4644375

[B62] Ohtani O. Ohtani Y. (2008). Lymph circulation in the liver. *Anat. Rec.* 291 643–652. 10.1002/ar.20681 18484610

[B63] Oikawa H. Masuda T. Sato S. Yashima A. Suzuki K. Sato S. (1998). Changes in lymph vessels and portal veins in the portal tract of patients with idiopathic portal hypertension: a morphometric study. *Hepatology* 27 1607–1610. 10.1002/hep.510270621 9620334

[B64] Ozeki M. Asada R. Saito A. M. Hashimoto H. Fujimura T. Kuroda T. (2019a). Efficacy and safety of sirolimus treatment for intractable lymphatic anomalies: a study protocol for an open-label, single-arm, multicenter, prospective study (SILA). *Regen Ther.* 10 84–91. 10.1016/j.reth.2018.12.001 30705924 PMC6348766

[B65] Ozeki M. Nozawa A. Kawamoto N. Fujino A. Hirakawa S. Fukao T. (2019b). Potential biomarkers of kaposiform lymphangiomatosis. *Pediatr. Blood Cancer* 66:e27878. 10.1002/pbc.27878 31207041

[B66] Ozeki M. Nozawa A. Yasue S. Endo S. Asada R. Hashimoto H. (2019c). The impact of sirolimus therapy on lesion size, clinical symptoms, and quality of life of patients with lymphatic anomalies. *Orphanet. J. Rare Dis.* 14 141. 10.1186/s13023-019-1118-1 31196128 PMC6567608

[B67] Ozeki M. Fukao T. (2019). Generalized lymphatic anomaly and gorham-stout disease: overview and recent insights. *Adv. Wound Care* 8 230–245. 10.1089/wound.2018.0850 31236308 PMC6589502

[B68] Paupert J. Sounni N. E. Noel A. (2011). Lymphangiogenesis in post-natal tissue remodeling: lymphatic endothelial cell connection with its environment. *Mol. Aspects Med.* 32 146–158. 10.1016/j.mam.2011.04.002 21549745

[B69] Petrova T. V. Makinen T. Makela T. P. Saarela J. Virtanen I. Ferrell R. E. (2002). Lymphatic endothelial reprogramming of vascular endothelial cells by the Prox-1 homeobox transcription factor. *EMBO J.* 21 4593–4599. 10.1093/emboj/cdf470 12198161 PMC125413

[B70] Pflicke H. Sixt M. (2009). Preformed portals facilitate dendritic cell entry into afferent lymphatic vessels. *J. Exp. Med.* 206 2925–2935. 10.1084/jem.20091739 19995949 PMC2806476

[B71] Platt A. M. Rutkowski J. M. Martel C. Kuan E. L. Ivanov S. Swartz M. A. (2013). Normal dendritic cell mobilization to lymph nodes under conditions of severe lymphatic hypoplasia. *J. Immunol.* 190 4608–4620. 10.4049/jimmunol.1202600 23530147 PMC3634097

[B72] Pupulim L. F. Vilgrain V. Ronot M. Becker C. D. Breguet R. Terraz S. (2015). Hepatic lymphatics: anatomy and related diseases. *Abdom Imaging.* 40 1997–2011. 10.1007/s00261-015-0350-y 25579171

[B73] Ritchie H. D. Grindlay J. H. Bollman J. L. (1959). Flow of lymph from the canine liver. *Am. J. Physiol.* 196 105–109. 10.1152/ajplegacy.1958.196.1.105 13617445

[B74] Rutkowski J. M. Moya M. Johannes J. Goldman J. Swartz M. A. (2006). Secondary lymphedema in the mouse tail: lymphatic hyperplasia, VEGF-C upregulation, and the protective role of MMP-9. *Microvasc. Res.* 72 161–171. 10.1016/j.mvr.2006.05.009 16876204 PMC2676671

[B75] Savetsky I. L. Ghanta S. Gardenier J. C. Torrisi J. S. Garcia Nores G. D. (2015). Th2 cytokines inhibit lymphangiogenesis. *PLoS One* 10:e0126908. 10.1371/journal.pone.0126908 26039103 PMC4454507

[B76] Schineis P. Runge P. Halin C. (2019). Cellular traffic through afferent lymphatic vessels. *Vascul Pharmacol.* 112 31–41. 10.1016/j.vph.2018.08.001 30092362

[B77] Schrage A. Loddenkemper C. Erben U. Lauer U. Hausdorf G. Jungblut P. R. (2008). Murine CD146 is widely expressed on endothelial cells and is recognized by the monoclonal antibody ME-9F1. *Histochem. Cell Biol.* 129 441–451. 10.1007/s00418-008-0379-x 18214516 PMC2756363

[B78] Schroder H. Marrugat J. Fito M. Weinbrenner T. Covas M. I. (2006). Alcohol consumption is directly associated with circulating oxidized low-density lipoprotein. *Free Radic. Biol Med.* 40 1474–1481. 10.1016/j.freeradbiomed.2005.12.014 16631537

[B79] Shin K. Kataru R. P. Park H. J. Kwon B. I. Kim T. W. Hong Y. K. (2015). TH2 cells and their cytokines regulate formation and function of lymphatic vessels. *Nat. Commun.* 6:6196. 10.1038/ncomms7196 25648335

[B80] Strauss O. Phillips A. Ruggiero K. Bartlett A. Dunbar P. R. (2017). Immunofluorescence identifies distinct subsets of endothelial cells in the human liver. *Sci. Rep.* 7:44356. 10.1038/srep44356 28287163 PMC5347010

[B81] Tamburini B. A. J. Finlon J. M. Gillen A. E. Kriss M. S. Riemondy K. A. Fu R. (2019). Chronic liver disease in humans causes expansion and differentiation of liver lymphatic endothelial cells. *Front. Immunol.* 10:1036. 10.3389/fimmu.2019.01036 31156626 PMC6530422

[B82] Tanaka M. Iwakiri Y. (2016). The hepatic lymphatic vascular system: structure, function, markers, and lymphangiogenesis. *Cell Mol. Gastroenterol. Hepatol.* 2 733–749. 10.1016/j.jcmgh.2016.09.002 28105461 PMC5240041

[B83] Tay M. H. D. Lim S. Y. J. Leong Y. F. I. Thiam C. H. Tan K. W. Torta F. T. (2019). Halted lymphocyte egress via efferent lymph contributes to lymph node hypertrophy during hypercholesterolemia. *Front. Immunol.* 10:575. 10.3389/fimmu.2019.00575 30972070 PMC6446103

[B84] Thomas S. N. Rohner N. A. Edwards E. E. (2016). Implications of lymphatic transport to lymph nodes in immunity and immunotherapy. *Annu. Rev. Biomed. Eng.* 18 207–233. 10.1146/annurev-bioeng-101515-014413 26928210 PMC5518935

[B85] Tirronen A. Vuorio T. Kettunen S. Hokkanen K. Ramms B. Niskanen H. (2018). Deletion of lymphangiogenic and angiogenic growth factor VEGF-D leads to severe hyperlipidemia and delayed clearance of chylomicron remnants. *Arterioscler. Thromb Vasc. Biol.* 38 2327–2337. 10.1161/ATVBAHA.118.311549 30354205

[B86] Torrisi J. S. Hespe G. E. Cuzzone D. A. Savetsky I. L. Nitti M. D. Gardenier J. C. (2016). Inhibition of inflammation and inos improves lymphatic function in obesity. *Sci. Rep.* 6:19817. 10.1038/srep19817 26796537 PMC4726274

[B87] Trutmann M. Sasse D. (1994). The lymphatics of the liver. *Anat. Embryol.* 291 201–209. 10.1007/BF00234299 7818092

[B88] Tugues S. Morales-Ruiz M. Fernandez-Varo G. Ros J. Arteta D. Munoz-Luque J. (2005). Microarray analysis of endothelial differentially expressed genes in liver of cirrhotic rats. *Gastroenterology* 129 1686–1695. 10.1053/j.gastro.2005.09.006 16285966

[B89] Weitman E. S. Aschen S. Z. Farias-Eisner G. Albano N. Cuzzone D. A. Ghanta S. (2013). Obesity impairs lymphatic fluid transport and dendritic cell migration to lymph nodes. *PLoS One* 8:e70703. 10.1371/journal.pone.0070703 23950984 PMC3741281

[B90] Wigle J. T. Harvey N. Detmar M. Lagutina I. Grosveld G. Gunn M. D. (2002). An essential role for Prox1 in the induction of the lymphatic endothelial cell phenotype. *EMBO J.* 21 1505–1513. 10.1093/emboj/21.7.1505 11927535 PMC125938

[B91] Wigle J. T. Oliver G. (1999). Prox1 function is required for the development of the murine lymphatic system. *Cell* 98 769–778. 10.1016/s0092-8674(00)81511-1 10499794

[B92] Witte C. L. Witte M. H. Dumont A. E. (1980). Lymph imbalance in the genesis and perpetuation of the ascites syndrome in hepatic cirrhosis. *Gastroenterology* 78(5 Pt 1), 1059–1068. 10.1016/0016-5085(80)90793-3 7380179

[B93] Witte C. L. Witte M. H. Dumont A. E. Cole W. R. Smith J. R. (1969). Protein content in lymph and edema fluid in congestive heart failure. *Circulation* 40 623–630. 10.1161/01.cir.40.5.623 5377204

[B94] Witte M. H. Dumont A. E. Cole W. R. Witte C. L. Kintner K. (1969). Lymph circulation in hepatic cirrhosis: effect of portacaval shunt. *Ann. Intern. Med.* 70 303–310. 5764506 10.7326/0003-4819-70-2-303

[B95] Witte M. H. Witte C. L. Dumont A. E. (1981). Estimated net transcapillary water and protein flux in the liver and intestine of patients with portal hypertension from hepatic cirrhosis. *Gastroenterology* 80 265–272. 10.1016/0016-5085(81)90713-7 7450417

[B96] Wong B. W. Wang X. Zecchin A. Thienpont B. Cornelissen I. Kalucka J. (2017). The role of fatty acid beta-oxidation in lymphangiogenesis. *Nature* 542 49–54. 10.1038/nature21028 28024299

[B97] Yamauchi Y. Ikeda R. Michitaka K. Hiasa Y. Horiike N. Onji M. (2002). Morphometric analysis of lymphatic vessels in primary biliary cirrhosis. *Hepatol. Res.* 24:107. 10.1016/s1386-6346(02)00019-0 12270739

[B98] Yamauchi Y. Michitaka K. Onji M. (1998). Morphometric analysis of lymphatic and blood vessels in human chronic viral liver diseases. *Am. J. Pathol.* 153 1131–1137. 10.1016/s0002-9440(10)65657-x 9777944 PMC1853063

[B99] Yokomori H. Oda M. Kaneko F. Kawachi S. Tanabe M. Yoshimura K. (2010). Lymphatic marker podoplanin/D2-40 in human advanced cirrhotic liver–re-evaluations of microlymphatic abnormalities. *BMC Gastroenterol.* 10:131. 10.1186/1471-230X-10-131 21059220 PMC2995474

[B100] Zhang F. Zarkada G. Han J. Li J. Dubrac A. Ola R. (2018). Lacteal junction zippering protects against diet-induced obesity. *Science* 361 599–603. 10.1126/science.aap9331 30093598 PMC6317738

[B101] Zhang Q. Liu J. Liu J. Huang W. Tian L. Quan J. (2014). oxLDL induces injury and defenestration of human liver sinusoidal endothelial cells via LOX1. *J. Mol. Endocrinol.* 53 281–293. 10.1530/JME-14-0049 25057109

[B102] Zheng M. Yu J. Tian Z. (2013). Characterization of the liver-draining lymph nodes in mice and their role in mounting regional immunity to HBV. *Cell Mol. Immunol.* 10 143–150. 10.1038/cmi.2012.59 23376862 PMC4003044

